# Type-I Interferon Signaling in Fanconi Anemia

**DOI:** 10.3389/fcimb.2022.820273

**Published:** 2022-02-07

**Authors:** Karima Landelouci, Shruti Sinha, Geneviève Pépin

**Affiliations:** ^1^ Département de Biologie Médicale, Université du Québec à Trois-Rivières, Trois-Rivières, QC, Canada; ^2^ Groupe de Recherche en Signalisation Cellulaire, Université du Québec à Trois-Rivières, Trois-Rivières, QC, Canada; ^3^ Department of Biotechnology, GITAM Institute of Technology, GITAM deemed to be University, Visakhapatnam, India

**Keywords:** Fanconi anemia, interferon, inflammation, DNA damage, cytosolic DNA, cGAS/STING, RIG-I

## Abstract

Fanconi Anemia (FA) is a genome instability syndrome caused by mutations in one of the 23 repair genes of the Fanconi pathway. This heterogenous disease is usually characterized by congenital abnormalities, premature ageing and bone marrow failure. FA patients also show a high predisposition to hematological and solid cancers. The Fanconi pathway ensures the repair of interstrand crosslinks (ICLs) DNA damage. Defect in one of its proteins prevents functional DNA repair, leading to the accumulation of DNA breaks and genome instability. Accumulating evidence has documented a close relationship between genome instability and inflammation, including the production of type-I Interferon. In this context, type-I Interferon is produced upon activation of pattern recognition receptors by nucleic acids including by the cyclic GMP-AMP synthase (cGAS) that detects DNA. In mouse models of diseases displaying genome instability, type-I Interferon response is responsible for an important part of the pathological symptoms, including premature aging, short stature, and neurodegeneration. This is illustrated in mouse models of Ataxia-telangiectasia and Aicardi-Goutières Syndrome in which genetic depletion of either Interferon Receptor IFNAR, cGAS or STING relieves pathological symptoms. FA is also a genetic instability syndrome with symptoms such as premature aging and predisposition to cancer. In this review we will focus on the different molecular mechanisms potentially leading to type-I Interferon activation. A better understanding of the molecular mechanisms engaging type-I Interferon signaling in FA may ultimately lead to the discovery of new therapeutic targets to rescue the pathological inflammation and premature aging associated with Fanconi Anemia.

## Introduction

Fanconi anemia (FA) is a rare recessive disease characterized by genome instability that results from a defect in the Fanconi DNA repair pathway. FA patients present aplastic anemia, congenital defects and cancer predisposition (Rodriguez and D’Andrea, 2017). Mechanistically, the FA pathway contributes to DNA damage recognition and repair, thus mutations in genes encoding FA pathway proteins lead to DNA damage accumulation and chromosome instability. As of today, 23 Fanconi and Fanconi-like DNA repair genes encoding either core complex proteins (FANCA, FANCB, FANCC, FANCE, FANCF, FANCG, FANCL and FANCM), ID2 complex proteins (FANCD2 and FANCI), and downstream proteins (FANCD1 (BRCA2), FANCJ (BRIP1), FANCN (PALB2), FANCO (RAD51C), FANCP (SLX4), FANCQ (ERCC4), FANCR (RAD51), FANCS (BRCA1), FANCT (UBE2T), FANCU (XRXX2), FANCV (MAD2L2/REV7), FANCW (RFWD3) and FANCY) have been identified. Findings from the last decade have unambiguously improved the management of the disease and prolonged the life of patients, and the many FA mouse models developed along the years were invaluable in getting a better understanding of the underlying cause of FA (Guitton-Sert et al., 2021).

## FA Proteins Are Involved in DNA Repair Processes

**Figure 1:**
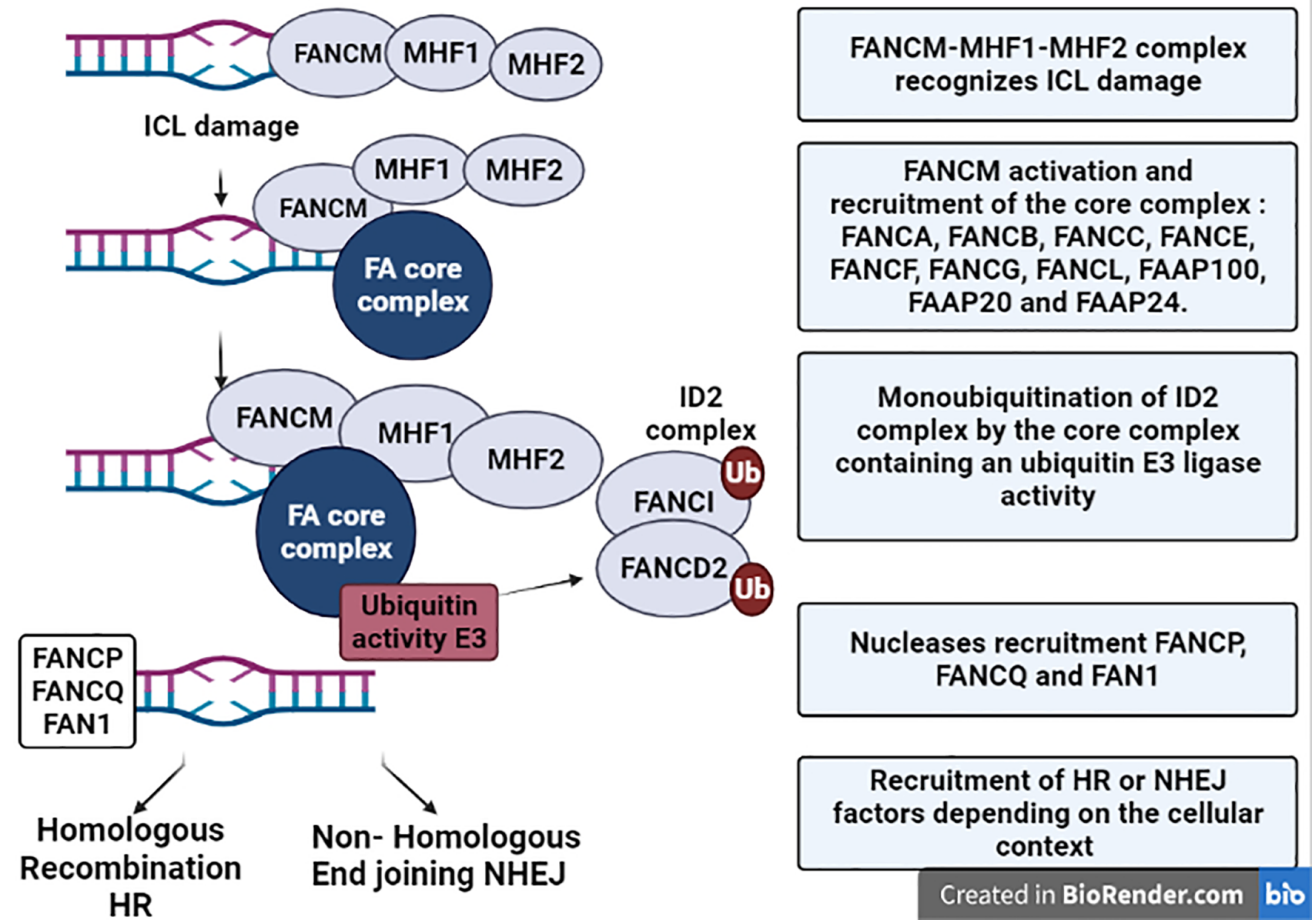
Schematic representation of ICLs repair by the Fanconi Anemia pathway. The protein complex composed of FANCM-MHF1-MHF2 is recruited to the chromatin upon recognition of ICL damage. Then, FANCM is activated and allows the recruitment of the core complex of the FA pathway composed of: FANCA, FANCB, FANCC, FANCE, FANCF, FANCG, FANCL, FAAP100, FAAP20 and FAAP24. The core complex contains an ubiquitin E3 ligase activity and is responsible for the monoubiquitylation of the ID2 complex composed of FANCI and FANCD2 proteins. Monoubiquitinylation of ID2 complex promotes the recruitment of the nucleases FANP, FANCQ and FAN1 and end resection at the DNA damage site. Depending on the cellular context, recruitment of downstream factors belonging to either Homologous Recombination (HR) or to Non-homologous End Joining (NHEJ) will finalize the repair.

The primary role attributed to FA proteins is the repair of inter-strand crosslinks (ICLs) DNA damage. ICLs, known to interfere with DNA replication and transcription, can be caused by exogenous sources such as chemotherapeutic agents like cisplatin and mitomycin C, or by endogenous cellular products like aldehydes. ICLs are repaired by the FA pathway (**Figure 1**), in the S-phase of the cell cycle (Knipscheer et al., 2009). The ICLs are first recognized by the FANCM–MHF1–MHF2 complex (Singh et al., 2010), followed by the activation of FANCM, which allows the recruitment of the core complex composed of ten proteins (FANCA, FANCB, FANCC, FANCE, FANCF, FANCG, FANCL, FA associated protein 100 (FAAP100), FAAP20 and FAAP24). The core complex possesses an ubiquitin E3 ligase that upon detection of the ICLs will monoubiquitinate FANCI and FANCD2 (ID2 complex) to form a higher order structure that initiates DNA repair (Tan et al., 2020; Tan and Deans, 2021). This latter step is essential to recruit nucleases such as FANCP (SLX4) – FANCQ (XPF) and FAN1, and to activate downstream repair factors that belong to the Homologous Recombination (HR) DNA repair complex: FANCJ – FANCN (PALB2) – FANCD1 (BRCA2) – FANCO (RAD51C) (Tan et al., 2020; Tan and Deans, 2021).

Besides the repair of ICLs, FA proteins are involved in other aspects of genome stability maintenance (Rodriguez and D’Andrea, 2017). For instance, the FA pathway stabilizes the replication fork during DNA synthesis to prevent accumulation of ssDNA or DNA-RNA hybrids called R-loops (Garcia-Rubio et al., 2015; Schwab et al., 2015; Xu et al., 2021). FANCJ promotes RPA phosphorylation during nucleotide excision repair (NER) and interacts with the mismatched repair (MMR) proteins MLH1 and MSH2 after UV irradiation to maintain genome stability (Guillemette et al., 2014). Furthermore, FANCC and FANCD2 cooperate with P53 to maintain the DNA damage-induced G2 checkpoint (Freie et al., 2004). FANCA and FANCB were both reported to catalyze single-strand annealing and strand exchange independently of their canonical role in the FA pathway (Benitez et al., 2018). The FA pathway is also linked to the maintenance of telomere integrity as FANCD2 mutated patient cells display several telomeric abnormalities (Joksic et al., 2012). How these different genome stabilization roles directly translate into all of the FA associated pathological symptoms, however, remains to be clarified. Interestingly, in previous studies FA-patients- or FA mouse models-derived cells with mutation in either FANCA, B, C or D, were shown to express higher levels of pro-inflammatory cytokines. In addition, FA cells (FANCA, B, C, D) were more susceptible to inflammatory stimulus (Rosselli et al., 1994; Dufour et al., 2003). These phenotypes were partly attributed to the dysregulated anti-oxidative response, as exemplified by the observation that FANCC knock-out cells express lower levels of anti-oxidative response genes and display higher levels of reactive oxygen species (ROS) (Zhang et al., 2007; Sejas et al., 2007).

**Figure 2:**
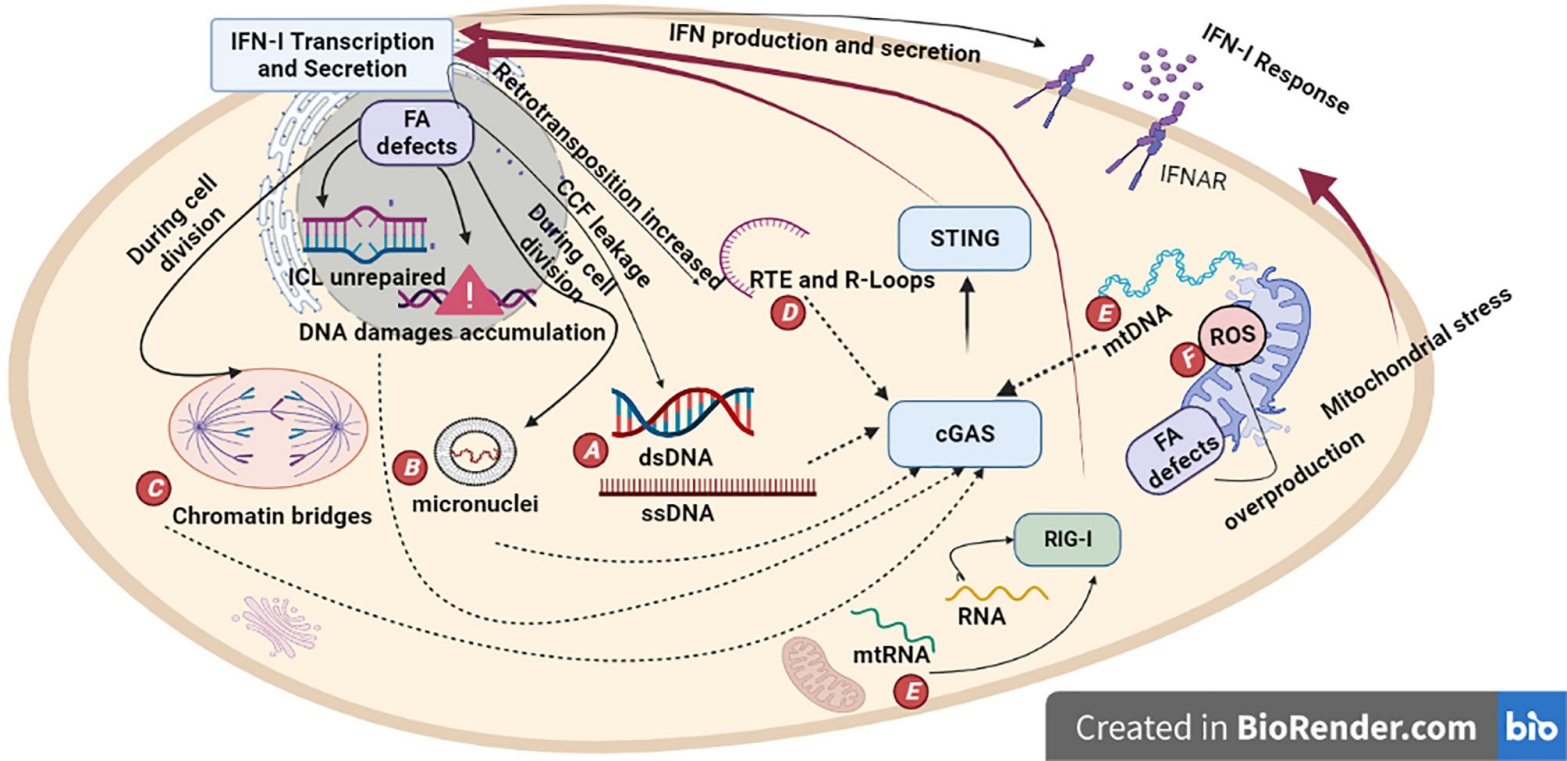
Proposed model depicting how defect in the FA pathway could induce pathological IFN-I production. FA proteins prevent genome instability by repairing DNA damage, repressing retrotransposition and allowing accurate cell division. In addition, FA proteins modulate anti-oxidative response and participate in mitochondria clearance by autophagy and mitophagy processes. Mutations in FA genes or loss of FA proteins contribute to DNA damage accumulation, genome instability, reactive oxygen species (ROS) production and defect in mitochondria’s clearance. Under these pathological conditions, many types of self-nucleic acids such as ssDNA and dsDNA **(A)**, micronuclei **(B)** chromatin bridges **(C)** retroelements (RTE) and R-loops **(D)**, mtDNA/RNA **(E)**, can activate type-I Interferon (IFN-I). This would occur by cGAS and RIG-I detection of cytosolic DNA and RNA molecules respectively and subsequent production of IFN-I. In addition, elevated levels of ROS might contribute to fuel the production of IFN-I by creating more DNA and mitochondria damage **(F)**.

During the last decade, a series of studies have demonstrated the complex intertwined relationship between DNA repair processes and immune responses. One key signaling axis involved in this association is the type-I Interferon (IFN-I) response known to play a critical role in the antiviral response (Taffoni et al., 2021). IFN-I response is essential to induce the transcription of genes involved in DNA repair and in nucleic acid degradation. Therefore, in the presence of defects in DNA repair processes or nucleic acid metabolism, cellular immune receptors can be activated by self-nucleic acids (Taffoni et al., 2021). Given that new evidence suggests that loss of function of FA proteins causes IFN-I production (Bregnard et al., 2016; Reislander et al., 2019; Heijink et al., 2019), this review explores how defect in the FA pathway might contribute to the accumulation of cytosolic nucleic acids and activation of IFN-I (**Figure 2**).

## Microbial Nucleic Acids Are Detected by Cellular Receptors

During infections, cells rely on a complex system of cellular receptors called Pattern Recognition Receptors (PRRs) that recognize pathogen-associated molecular patterns (PAMPs) to elicit an immune response that will allow the cell to defend itself against the invaders and alert neighboring immune cells of the ongoing infection. Microbial nucleic acids are among these PAMPs and are detected by different families of PRRs including Toll-like receptors (TLR), RIG-I like receptors (RLR) and cyclic GMP-AMP synthase (cGAS) (Kretschmer and Lee-Kirsch, 2017). TLRs that detect nucleic acids (TLR3-7-8 detect RNA and TLR9 detects DNA) are found predominantly within endosomes and are mostly expressed in specialized immune cells (monocytes, macrophages, dendritic cells and B lymphocytes) (Schlee and Hartmann, 2016). On the contrary, RLR and cGAS are expressed ubiquitously and are located within the cytoplasm of cells. While RIG -I and MDA-5 activate IFN-I upon sensing short and long cytosolic dsRNA respectively (Reikine et al., 2014), cGAS detects dsDNA (or ssDNA fold into dsDNA) and DNA : RNA hybrids (Ablasser et al., 2013; Mankan et al., 2014; Schlee and Hartmann, 2016). Upon sensing their specific nucleic acids, these PRRs activate an immune response that leads to the production of inflammatory cytokines and type I-Interferons (IFN-I). Cells and organisms rely on this response to fight microbial infections.

## Sensing of Nucleic Acids by Cellular Receptors Activates the Type-I Interferon Response

At the molecular level, RIG-I and MDA5 receptors signal through the Mitochondrial antiviral-signaling adaptor protein (MAVS) to allow the translocation and activation of Interferon Regulatory Factor 3 (IRF3) that binds to regulatory elements on IFN-I genes to induce their transcription and translation (Schlee and Hartmann, 2016). Binding of IFN-I in an autocrine or paracrine manner activates Janus Kinase 1 (JAK1) and Tyrosine Kinase 2 (TYK2), which then phosphorylate the Signal Transducer and Activator of Transcription 1 (STAT1) and 2 (STAT2) transcription factors to allow the transcription of thousands of genes collectively referred to as Interferon Stimulated Genes (ISGs) (Platanias, 2005; Rusinova et al., 2013). These genes are known to alter cellular metabolism, arrest the cell cycle, induce apoptosis and favor the clearance of damaged cells by immune cells (Rusinova et al., 2013).

Upon cytosolic DNA detection, cGAS catalyzes the production of the cyclic dinucleotide GMP-AMP (cGAMP) from ATP and GTP (Ablasser et al., 2013). Then, cGAMP acts as a secondary messenger to activate the Stimulator of Interferon Genes (STING), inducing its conformational change and subsequent activation (Ablasser et al., 2013; Wu et al., 2013; Sun et al., 2013). In its activated form, STING interacts with Tank-binding Kinase I (TBK1) and the IKK complex to activate them, which leads to STING phosphorylation by TBK1, then recruitment and activation of IRF3 followed by the release of Nuclear Factor-kappaB (NF-κB), ultimately promoting the production of IFN-I and other inflammatory cytokines (Balka and De Nardo, 2021). During acute immune responses, IFN-I activation is usually beneficial to the host by providing transcriptional changes that allow the orchestration of an efficient response. However, when this activation persists, it becomes detrimental and is associated with chronic diseases (Crow and Stetson, 2021).

## Cytosolic Accumulation of Self- Nucleic Acids Allows Activation of RLRs and cGAS

During the last decades, these PRRs were also shown to alert the host of the loss of cellular integrity. Indeed, RLRs and cGAS can also detect self-nucleic acids in non-infectious contexts (Ablasser and Hur, 2020). To prevent unwanted inflammation, nucleic acids are usually protected from recognition by PRRs by localizing inside organelles or by being coated with proteins. However, under certain cellular conditions, these barriers are broken and self-nucleic acids are released into the cytosol. Fortunately, to avoid persistent immune activation, the genome encodes many DNases and RNases to degrade cytosolic nucleic acids (Santa et al., 2021). Whether or not PRRs will get activated by self-nucleic acids is thus dependent on the balance between the activity of DNases and RNases versus the amount of nucleic acids released into the cytosol at a given point. Thus, defect in either of these processes can result in PRR activation. This model has been validated by *in vitro* experiment, mouse models and clinical data. Indeed, higher levels of circulating IFN-I in mouse models and in patients with mutations in gene encoding DNases and RNAses were detected (Crow and Stetson, 2021). In addition to this, organelles and genome integrity also play a critical part in safeguard mechanisms. For instance, the nuclear envelope (NE) protects the genome inside the nucleus, maintains its dynamic shapes and regulates nuclear exchanges with the cytosol. Mutations in NE gene’s structure result in the loss of NE integrity and promote genome instability and the release of nucleic acids into the cytosol (Gauthier and Comaills, 2021). Similarly, loss of mitochondria integrity causes mitochondrial (mt) DNA release into the cytosol and immune activation (Lepelley et al., 2021).

## Constitutive Activation of IFN-I in DNA Repair and DNA Metabolism Related-Diseases

Constitutive IFN-I activation is detected in different autoinflammatory syndromes such as Aicardi-Goutières syndrome (AGS) and Ataxia-telangiectasia (AT) syndrome. AGS is caused by mutations in genes involved in nucleic acid metabolism such as those encoding the Three Prime Repair Exonuclease 1 (TREX1) (Rice et al., 2007), the RNASEH2 (Crow et al., 2006) and the triphosphohydrolase SAMHD1 (Coquel et al., 2018). In mouse models of AGS, cytosolic DNA accumulation activates IFN-I. Notably, genetic depletion of cgas (Gray et al., 2015), sting (Gall et al., 2012), irf3 or ifnar (Stetson et al., 2008) inhibits the IFN-I response and relieves autoinflammatory symptoms, confirming the important contribution of IFN-I to AGS pathology. AT syndrome is, on the other hand, more closely related to FA pathophysiology and also relies on chronic STING activation (Hartlova et al., 2015; Aguado et al., 2021). AT is caused by mutations in the Ataxia-Telangiectasia-Mutated kinase (ATM), a master regulator of DNA repair. In fact, loss of ATM activity results in cytosolic DNA accumulation due to genomic instability (Hartlova et al., 2015). This can be explained by the crucial role of ATM in coordinating the cellular response to double-strand DNA breaks (DSBs). AT patients-derived cells and cells isolated from Atm^-/-^ mice harbor single-strand (ss)DNA in their cytoplasm and an elevated IFN-I signature. Genetic depletion of sting inhibits the pathological IFN-I response in those cells (Hartlova et al., 2015). As AT shares redundant phenotypes with FA, including high genomic instability, dysregulated immune responses, neurodegeneration, premature aging and predisposition to cancers and leukemias, and considering the preponderant role of IFN-I in AT, it is reasonable to suspect that IFN-I also contributes to the pathophysiology of FA.

## FA Deficient Cells Display Cytosolic DNA Accumulation

FA is a genome instability syndrome. The literature suggests that FA deficient cells display accumulation of cytosolic DNA, loss of nuclear integrity and a defect in mitochondria clearance (Naim and Rosselli, 2009; Bregnard et al., 2016; Shyamsunder et al., 2016; Sumpter et al., 2016; Reislander et al., 2019; Heijink et al., 2019). As mentioned above, these processes contribute to tipping the scale toward PRR activation and IFN-I production. Indeed, with their reported roles in DNA repair and cell division processes, FA proteins prevent genome instability and cytosolic DNA accumulation. In other genome instability syndromes, higher levels of IFN-I have been linked to neurological disorders, growth delays, premature ageing and skin pathologies among others.

## Accumulation of Cytosolic ssDNA in Cells With Defect in DNA Repair Mechanisms

Following the elucidation of how ATM deficiency activates the IFN-I response (Hartlova et al., 2015), several other examples of how DNA-repair defects trigger unwanted immune activation through the release of cytosolic ssDNA were reported (Bregnard et al., 2016; Chen et al., 2017; Erdal et al., 2017; Coquel et al., 2018; Gratia et al., 2019; Heijink et al., 2019; Guan et al., 2021). During repair, the ubiquitylated ID2 complex recruits the scaffold protein FANCP/SLX4 which serves as a docking site for the endonucleases that cleave the damaged DNA to create a DSB that will be repaired by homologous recombination (HR). These endonucleases generate ssDNA overhangs that are coated and protected from degradation by the replication protein A (RPA) and the recombinase RAD51. Notably, mutations in proteins controlling long-range DNA resection following double-strand break repair, like the endonuclease MUS81, the Bloom syndrome helicase (BLM) and the exonuclease 1 (EXO1) result in cytosolic ssDNA accumulation and cGAS-STING-IFN-I activation - a phenotype negatively controlled by the TREX1 exonuclease, which degrades these ssDNA (Ho et al., 2016; Erdal et al., 2017; Guan et al., 2021). Furthermore, knockdown of RPA or RAD51 leads to unprotected ssDNA which favors IFN-I production by the cGAS-STING pathway (Wolf et al., 2016). This phenomenon is seen in at least one other DNA repair mechanism. Indeed, defects in the mismatch repair pathway (MMR) have recently been shown to induce an uncontrolled activity of the EXO1 nuclease, leading to ssDNA release and RPA exhaustion causing cGAS-STING activation (Guan et al., 2021). Given that FA proteins (FANCJ, FANCD2 and BRCA1) were suggested to interact with the MHL1 MMR factor during mismatch repair (Peng et al., 2014), this could be another mechanism that leads to IFN-I production in FA. Together, these data suggest that many factors like nucleases or signaling proteins are implicated in the amount of ssDNA released into the cytosol and thus in the activation of IFN-I. Indeed, in lymphomas, cytosolic DNA presence is dependent on the downstream signaling of Ataxia Telangiectasia and Rad3-related protein (ATR) and to a lesser extent on ATM (Lam et al., 2014). Given that FA deficient cells have defect in DNA repair mechanisms, cytosolic DNA could accumulate and be detected by cGAS to activate the production of IFN-I, as schematized in **Figure 2A**.

## The Presence of Micronuclei Correlates With cGAS Self-DNA Sensing

In addition to ssDNA fragments release, cGAS-STING-dependent IFN-I response correlates with micronuclei generation upon chromosomes mis-segregation (Harding et al., 2017; Mackenzie et al., 2017; Gratia et al., 2019), as represented in **Figure 2B**. In a seminal study, Harding et al. demonstrated that micronuclei formation requires cell division and a functional Non-Homologous End Joining (NHEJ) DNA repair pathway. Hence, inhibition of either NHEJ or cell cycle progression prevents cGAS activation (Harding et al., 2017). However, how the DNA packaged in micronuclei activates cGAS is still under debate. Some plausible explanations include nuclear envelope rupture and then cGAS detection of the released cytosolic DNA (Mackenzie et al., 2017). Alternatively, if nuclear cGAS is packaged within micronuclei, cGAS activation after the nuclear envelope ruptures would require its reactivation because nuclear cGAS displays reduced enzymatic activity (Harding et al., 2017; Gentili et al., 2019; Volkman et al., 2019). There might also be a threshold that needs to be reached/crossed since a recent study suggested that cGAS itself contributes to autophagic clearance of micronuclei (Zhao et al., 2021). Nonetheless, other less-visible DNA structures in cells displaying micronuclei could also contribute to the IFN-I activation. This hypothesis is supported by a new study in which the authors claim that DNA bridges and not micronuclei are responsible for cGAS activation (Flynn and P.D. and Mitchison, 2021). However, whether cGAS is directly located along DNA bridges and how this positioning would allow cGAS activation remains to be elucidated. New data also proposes that the inclusion of cGAS in micronuclei can be modulated by the end-resection machinery, as pharmaceutical inhibition of end-resection or depletion of its components NBS1 and the DNA endonuclease CtlP favor cGAS localization in micronuclei (Abdisalaam et al., 2020). Of interest, micronuclei were also observed in cancer cells in which FANCD1 (BRCA2) expression was abrogated (Heijink et al., 2019; Reislander et al., 2019). Interestingly, depletion of BRCA2 renders the cells more sensitive to Poly [ADP-ribose] polymerase (PARP) inhibitors (Reislander et al., 2019) and to TNFα cytotoxicity in a cGAS dependent manner (Heijink et al., 2019). Given that cancer cells have already accumulated mutations, whether this would occur in FA patients-derived cells remains to be investigated.

## The cGAS-STING-IFN-I Axis Might Contribute to the HR-NHEJ DNA Repair Switch

FA cells are characterized by a propension to repair their DSBs using the more error-prone and mutagenic NHEJ rather than HR, which utilizes end-resection during the process (Adamo et al., 2010; Eccles et al., 2018). Accordingly, FA proteins cooperate with the kinase ATM to stimulate end-resection repair (Cai et al., 2020). Thus, loss of either ATM or FA proteins shifts the repair mechanism toward NHEJ. Surprisingly, cGAS and STING were both reported to inhibit HR DNA repair, albeit using different mechanisms (Liu et al., 2018; McLaughlin et al., 2020). STING-driven IFN-I production negatively regulates the transcription of genes implicated in HR repair like FANCD2 and other BRCA genes (including FANCC and FANCE). Importantly, treatment with the Jak 1/2 inhibitor, Ruxolitinib, was sufficient to rescue the HR defect, suggesting that their transcriptional repression is a result of IFN-I signaling (McLaughlin et al., 2020). Additionally, cGAS also prevents HR repair by a distinct mechanism. cGAS was shown to translocate to the nuclear compartment upon treatment with DNA damage agents, where cGAS inhibits HR by preventing the formation of PARP1-Timeless complex. However, it is not clear what the contribution of cGAS catalytic activity is in this model since the experiments were performed in U2OS cells which lack expression of the downstream signalling STING protein (Deschamps and Kalamvoki, 2017). Given this newly discovered relationship, it would be of interest to determine if the depletion of cGAS-STING or IFNAR in FA deficient cells would redirect DNA repair toward HR.

## cGAS Independent Activation of IFN-I Response

As discussed above, cGAS detection of cytosolic DNA is a major route to produce IFN-I upon DNA damage. However, it is worth noting that several other mechanisms were also described. For instance, studies showed that DNA damage induced by etoposide or by doxorubicin instigates an ATM-dependent but cGAS-independent immune response (Luthra et al., 2017; Dunphy et al., 2018). In addition, DNA-dependent protein kinase (DNA-PK) was characterized as another DNA sensor contributing to IFN-I (Ferguson et al., 2012), and more recently, DNA-PK has been suggested to induce a cGAS-STING-independent IFN-I induction upon DNA damage in humans but not in mice, highlighting species differences in immune activation following DNA breaks (Burleigh et al., 2020). Further, DNA-PK antagonizes cGAS activation through inhibition of its catalytic activity. In accordance with a role as a negative regulator of cGAS, cells lacking DNA-PK display an abnormal antiviral response and patients with mutations in the gene encoding DNA-PK suffer from autoimmunity (Sun et al., 2020). Given that cGAS expression itself is induced by IFN-I, cGAS-independent activation of IFN-I is expected to sensitize cells to cGAS detection of cytosolic DNA including R-loops, which are also released in the context of FA (Mankan et al., 2014; Chatzidoukaki et al., 2021). This positive loop might explain why healthy cells do not necessarily engage cGAS upon DNA damage while cells with defect in DNA repair with a basal IFN-I activation have a lower threshold for cGAS engagement (Pepin et al., 2017a; Pepin et al., 2017b).

## Micronuclei Formation and Chromatin Bridges Are a Hallmark of Cells With Defective Cell Division

Unrepaired DNA damage during cell division can result in chromatin bridges (**Figure 2C**) and micronuclei formation (**Figure 2B**) and thus cause aneuploidy and genome instability. As mentioned in the previous section, these structures containing genomic DNA can activate cGAS (Harding et al., 2017; Mackenzie et al., 2017; Gratia et al., 2019; Flynn and P.D. and Mitchison, 2021). Besides their roles in DNA repair, evidence suggests that the FA proteins (FANCA, C, D2, D1, G, I, M) also regulate chromosome separation and cytokinesis (Nakanishi et al., 2007; Naim and Rosselli, 2009; Vinciguerra et al., 2010; Kim et al., 2013; Nalepa et al., 2013; Zou et al., 2013). As such, an RNAi screen targeting 16 members of the FA complex showed that FA proteins prevent multinucleation of Taxol-treated cells. The relevance of this finding was confirmed by the fact that multinucleation was only observed in FA patients-derived fibroblast cells treated with Taxol but not in healthy patient cells (Nalepa et al., 2013). In line with their role in cell division, many FA proteins, including FANCA and FANCG, localize to the centrosome or elsewhere on mitotic chromosomes to prevent the formation of ultrafine chromatin bridges (Nakanishi et al., 2007; Naim and Rosselli, 2009; Vinciguerra et al., 2010; Nalepa et al., 2013; Zou et al., 2013). Accordingly, FANCA interacts with the spindle assembly checkpoint to prevent premature anaphase onset and sister chromatids segregation (Nalepa et al., 2013). FANCA is also responsible for centrosome integrity since overexpression of a mutant form leads to an aberrant number of centrosomes (Kim et al., 2013). As micronuclei and chromatin bridges activate cGAS, the defects in chromosome segregation and cytokinesis, observed in FA cells, are logically expected to result in IFN-I production (**Figures 2B, C**).

## Derepression of Retroelements Results in Cytosolic Self-Nucleic Acids Accumulation

Retroelements (RTE) such as LINE1 are ancient sequences that occupy around 40% of the genome. They can be transcribed into RNA and retrotranscribed into DNA forming RNA : DNA hybrids. Because of their retrotransposition activity, RTE contributes to genome instability and their expression correlates with ageing and induces IFN-I. Repressing RTE is another function of FA proteins. BRCA1 and the proteins of the FA core complex contribute to the broad repression of RTE, albeit using distinct mechanisms (Mita et al., 2020) (**Figure 2D**). In addition, Laguette’s group showed that a mutation in SLX4 (FANCP) or loss of FANCD2 or MUS81 increases retrotransposition and IFN-I activation (Bregnard et al., 2016). Accordingly, treatment of their cells with the retro-transcriptase inhibitor (RTi) Tenofovir abrogates both retrotransposition and IFN-I response. They went on to show that IFN-I production was dependent on the cGAS-STING pathway using specific shRNAs (Bregnard et al., 2016). In cells derived from AGS mouse models, RTE induce both the cGAS-STING (Achleitner et al., 2017) and the RIG-I signaling axes (Zhao et al., 2018). However, the use of RTi *in vivo* did not impact the systemic IFN-I response observed in AGS mouse models while still preventing retrotransposition in the animal cells (Achleitner et al., 2017). Lack of therapeutic effect of RTi in AGS mouse models might be attributed to the difference in the cell population studied *in vitro* vs the cell populations implicated in the pathophysiology of the disease. Thus, the relevance of RTE-driven IFN-I response in FANCP (SLX4) mutated cells to FA pathophysiology warrants further investigation.

## FA Deficient Cells Display Senescent Features

Cellular senescence is defined as a stable cell-cycle arrest. It occurs naturally during embryogenesis and can be triggered in different contexts such as replicative senescence, oncogene-induced senescence or following cellular stress. Apart from developmental senescence, the other senescence categories usually rely on the p53/p21 axis and the pRB/p16 axis, the former usually triggered by DNA damage and genome instability. During senescence, cells undergo major phenotypic and molecular changes including structural changes as well as secretion of inflammatory molecules, proteases and signalling molecules that are collectively referred to as the senescence associated secretory phenotype (SASP). FA was recently suggested to be a premature ageing disease based on the several senescence characteristics observed in FA cells and patients (Helbling-Leclerc et al., 2021). In many FA mouse models, including Fancd2^-/-^, Fancc^-/-^ and Fanca^-/-^, an exacerbated p53/p21 axis has been documented, contributing to bone marrow failure (Ceccaldi et al., 2012). This hyperactive p53/p21 axis has also been observed in FA patient-derived cells (Ceccaldi et al., 2012). Mechanistically, activation of the p53/p21 axis induces high levels of S-phase inhibitors which stop FA cell’s growth leading them to arrest in G0/G1 due to replicative stress (Ceccaldi et al., 2012). Another study reported that FANCD2 proteins prevent the progression of oncogene-induced senescence, meaning that its depletion favors premature senescence (Helbling-Leclerc et al., 2019). In parallel, Tumor Growth Factor β1 (TGFβ1), a known inducer of the cyclin-dependent kinase (CDK) inhibitor p21, is overproduced in cells isolated from the bone marrow of Fancd2^-/-^ mice (Zhang et al., 2016), thus inhibiting hematopoietic stem cell (HSC) cycling and precipitating bone marrow failure by incapacitating the HSC to repopulate progenitor and differentiated blood cells. Notably, specific deletion of either TGFβ1 or p53 rescues bone marrow failure by stimulating HSC cycling (Ceccaldi et al., 2012; Zhang et al., 2016). To overcome this cycling blockade, FA cells overexpressed the transcription factor MYC, which causes proliferative pressure and more DNA damage (Rodriguez et al., 2021). However, HSC cycling stimulation in FA mouse models leads to HSC exhaustion and bone marrow aplasia, indicating that the search for a better target in FA should continue. In this context, IFN-I signalling also promotes HSC cycling at the cost of more DNA damage in Fanca^-/-^ mice (Walter et al., 2015). These studies confirm the close relationship between FA and cell cycle arrest, but whether it is best characterized by senescence remains to be demonstrated. This idea is however supported by the fact that overexpression of SIRT6 histone deacetylases, previously reported to inhibit RTE transcription in Fanca^-/-^ or Fancd2^-/-^ mouse models, rescues stem cell exhaustion by reducing pathological inflammation (Li et al., 2018; Simon et al., 2019). Interestingly, a study showed that RTE are derepressed in late senescence, allowing them to be detected by the cGAS-STING pathway, thus contributing to the IFN-I response (De Cecco et al., 2019).

## Accumulation of Cytosolic DNA During Senescence Drives IFN-I Activation

Several reports have established that the cGAS-STING axis is activated in stress-induced senescence (e.g., replicative, oxidative and oncogene-induced senescence). The groups of Chen (Yang et al., 2017), Berger (Dou et al., 2017) and Hornung (Gluck et al., 2017) demonstrated that cytosolic DNA detection by cGAS is a critical step in the amplification of the SASP. The faster spontaneous immortalization of mouse embryonic fibroblast (MEF) cells deficient in cGAS was the first hint to its role in senescence (Yang et al., 2017). Initially associated to its role in SASP production, it was later discovered that cGAS also has a role in slowing down the replication fork and thus cell replication (Chen et al., 2020). Nonetheless, cGAS engagement clearly contributes to the SASP. Micronuclei, cytosolic chromatin fragments (CCF) and RTE are all suggested to be a source of cGAS activation in this context (**Figures 2A, B, D**). Similar to what was found for the role of NBS1 in controlling cGAS localization in micronuclei, cGAS detection of CCF has been suggested to depend on HMGB2 and topoisomerase 1-DNA covalent cleavage complex (TOP1cc) (Zhao et al., 2020). Activation of cGAS in a senescent context might also result from an imbalance between the production of CCF and the cell capacity to degrade these CCF. Accordingly, DNASE2 and TREX1 usually prevent CCF accumulation; however, during senescence, their expression is reduced and CCF accumulates and activates cGAS (Takahashi et al., 2018). In the disease context of AT syndrome, which presents striking phenotypic similarities to FA, cGAS is engaged during the senescence of AT patients olfactory neurosphere-derived cells and brain organoids, which display micronuclei. Importantly, cGAS and STING inhibition in this model prevents astrocyte senescence and protects brain organoids from neurodegeneration (Aguado et al., 2021). Given that FA syndrome was recently described as a ‘*senescence syndrome’* (Helbling-Leclerc et al., 2021), the premature ageing observed in FA might also be an important source of IFN-I production.

## Constitutive Reactive Oxygen Species Production in FA Fuels DNA Damage

Reactive oxygen species (ROS), mainly but not exclusively produced by mitochondria, have many functions in cells. They serve as signalling molecules and they oxidize biological molecules. The discovery that lymphocytes from FA patients grew better and generated less DNA breaks in low oxygen concentration was the first hint that the oxidative response was aberrant in these cells (Joenje et al., 1981; Schindler and Hoehn, 1988). This was later confirmed by the observation that cells isolated form Fancc^-/-^ mice overproduced ROS upon inflammatory stimulation (Sejas et al., 2007). ROS are indeed produced upon stimulation with TNFα, a pro-inflammatory cytokine overproduced by FA cells *in vitro* and *in vivo* (Rosselli et al., 1994; Dufour et al., 2003; Zhang et al., 2007). ROS overproduction has many effects on cells, one of which is the generation of DNA damage through the oxidation of DNA bases. Guanines are hypersensitive to oxidation and result in 8-oxoguanine (8-oxoG), the most abundant DNA lesion formed after oxidative exposure. Accordingly, unrepaired 8-oxoG lesions have been observed in the peripheral tissues of FA patients harboring mutations in FANCA, FANCC and FANCD2 genes (Du et al., 2012). These lesions correlate with a lower expression of antioxidant genes, contributing to the excessive oxidation detected in cells from FA patients (Du et al., 2012). Fancc^-/-^ mice also display higher levels of 8-oxoG DNA and yH2AX positive cells basally compare to wt mice. Interestingly, 8-oxoG DNA is resistant to degradation by TREX1 exonuclease (Gehrke et al., 2013). ROS can also cause mtDNA damage, and a new study suggests that mtRNA is released and then detected by RIG-I, a potent activator of IFN-I, upon mtDNA double-strand breaks (Tigano et al., 2021) as illustrated in **Figure 2E**. To our knowledge, RLR receptors have not been implicated in FA yet. In addition to mtRNA release, the RAD51C (FANCO) protein was recently shown to protect mitochondrial replication fork from degradation by the MRE11 mitochondrial nuclease during oxidative stress. In FA-derived patient cells with RAD51C mutation, this protection is lost and thus cytosolic mtDNA, derived from nascent mtDNA cleaved by MRE11, activates the cGAS-STING pathway (Luzwick et al., 2021). Importantly, this process has been reported to rely on FANCD2 and downstream FANC factors but to be independent of the FA core complex, highlighting the importance of understanding the specific roles of FA proteins (Luzwick et al., 2021). Thus, high levels of ROS might contribute to the creation of a pathological feedback loop by creating more DNA damage, ssDNA and mtDNA/RNA release and micronuclei formation that will induce IFN-I production which in turn would stimulate the production of more ROS (**Figure 2F**).

## Constitutive IFN-I Signaling in Cells With Defects in Mitochondria Clearance

Damaged mitochondria can be removed from the cell by classical autophagy, during which a membrane is formed around it and then it is fused to a lysosome, or by mitophagy, a process based on the specific recognition of mitochondrial factors by mitophagy receptors. Many FA proteins, including FANCC, FANCA and BRCA2, are required for two types of selective autophagy, the selective clearance of viruses (virophagy) and mitophagy. Consequently, a lack of these FA proteins impairs mitochondria clearance resulting in cellular accumulation of damaged mitochondria (**Figures 2E, F**) (Shyamsunder et al., 2016; Sumpter et al., 2016). FANCC directly interacts with PARKIN, which is involved in the clearance of damaged mitochondria and the reduction of ROS production. Importantly, using a specific variant of FANCC, c.67delG, it was shown that the DNA repair function and the mitophagy clearance function of FANCC are uncoupled, which suggests that the role of FANCC in mitophagy is relevant to FA pathophysiology (Sumpter et al., 2016). Damaged mitochondria release Danger Associated Molecular Patterns (DAMPs), including mtDNA, that are detected by innate immune receptors. Accordingly, mitochondrial stress arising from ATM inhibitor treatment, deletion of the nucleoid protein TFAM or Herpesvirus infection, causes mtDNA release and IFN-I response (West et al., 2015; Hu et al., 2021). The underlying mechanism of DNA release is not fully understood, although it was demonstrated that mitochondrial herniation mediated by BAK and BAX could be responsible for the DNA release (McArthur et al., 2018).

The fact that FA mouse models do not recapitulate the full spectrum of FA pathophysiology has hampered our understanding of this disease for a long time. However, the discovery that FA mice subjected to environmental stress such as tail bleeding or inflammatory stimulus undergo complete bone marrow failure (Walter et al., 2015) changed our understanding of the disease. It became clear that FA mice, in their highly controlled living conditions, did not reflect the complexity of a human life. Researchers working on Parkinson’s using mice genetically deficient in PINK1/Parkin faced the same challenge. In a seminal paper, Slitter and colleagues elegantly showed that if you submit these mice to exercise training, they developed cGAS-STING dependent symptoms highly resembling Parkinson’s disease. They went on to characterize the underlying molecular mechanism and demonstrated that faulty mitophagy was causing mtDNA release and cGAS activation (Sliter et al., 2018). Critically, genetic depletion of Sting in their model abolishes the pathological inflammation and prevents dopaminergic neuron death. Damaged mitochondria and STING-dependent neuronal cell death were also observed in Amyotrophic lateral sclerosis, suggesting that STING activation is a recurrent cause of neurodegeneration (Yu et al., 2020). Altogether, these findings highlight the complex interplay between DNA damage, immune response and cellular homeostasis.

## Targeting IFN-I in FA and Other Genome Instability Syndromes

To our knowledge, there are currently no clinical trials involving the use of Jak 1,2 inhibitors or IFN-I receptor blocking antibodies in FA’s closely-related syndromes such as AT. However, medical benefits were observed in diseases involving gain-of-function of STING and STAT1 (Forbes et al., 2018; Crow et al., 2021). In these syndromes, patients usually display measurably high levels of IFN-I. In AGS, treatment with the Janus kinase inhibitor Baricitinib confers a significant reduction in skin lesions and a possible improvement in neurologic symptoms (Vanderver et al., 2020). Although these data are encouraging, it is worth noting that IFN-I signaling could still be measured in the blood of patients suggesting that these inhibitors have limited efficacy (Crow et al., 2021). This lack of efficacy might be explained by the possibilities that the dosage needed to reach a stronger inhibition is prevented by the toxicity-associated side-effects of the drug or that the drug does not reach all cell populations equally. Nonetheless, these results suggest that IFN-I inhibition could relieve central nervous system (CNS) abnormalities in FA patients (Stivaros et al., 2015; Aksu et al., 2020).

Bone marrow failure is one of the most frequent symptoms for FA patients. IFN-I signalling is linked to bone marrow (BM) suppression. For instance, treatment of HCV patients with PEG- IFNα2a mediates BM suppression (King et al., 2015). Interestingly, BM failure has also been documented in case of chronic viral infection suggesting that persistent low levels of IFN-I might be sufficient to promote BM failure (Binder et al., 1997). In line with what is observed in FA, major suppressive impact of IFN-I on bone marrow will often occur following secondary stress (de Bruin et al., 2013). While worth testing, long-term treatment to inhibit IFN-I might also worsen cytopenia as observed upon treatment with Jak inhibitor for patients with myelofibrosis (Bose and Verstovsek, 2020). Whether this side-effect would be observed in FA patients needs further investigation.

FA patients display a high prevalence of many types of cancer. Given the opposing roles of IFN-I in cancer, the use of Jak inhibitor might worsen FA patient’s prevalence to cancer. On one side, IFN-I has critical role in cancer anti-tumor immunity, meaning it stimulates the ability of immune cells to destroy cancer cells. On the other side, prolonged IFN-I signaling can contribute to immune exhaustion and stimulates tumor growth (Boukhaled et al., 2021).

## Discussion

The last decade of research has recognized the crosstalk between DNA damage responses and inflammation, and emphasized its relevance in the study of syndromes caused by mutations in genes that maintain genome stability. The first demonstration of the implication of IFN-I in FA cells by Laguette’s group opened new and exciting avenues to determine its importance in FA pathophysiology. Currently, a strong correlation between IFN-I signaling and defect in BRCA2 related genes has been made in several cancer cell lines (Heijink et al., 2019; Reislander et al., 2019). An important question arising from these studies is how transposable to primary cells are the findings obtained using cancer cells? We previously reported that primary cells were more refractory to IFN-I induction upon DNA damage than their immortalized counterparts (Pepin et al., 2017a; Pepin et al., 2017b). In primary cells, prolonged cell cycle arrest following checkpoint activation prevents accumulation of self-nucleic acids (Chen et al., 2020). To investigate this question, it would be important to analyze primary tissues and a diversity of cell population from FA patients when possible and mouse models. However, when analyzing tissue from mouse models, non-canonical roles of proteins will need to be considered. Recent studies indicating that cGAS promotes genome instability (Liu et al., 2018; Jiang et al., 2019; Banerjee et al., 2021) and that STING modulates ROS metabolism (Hayman et al., 2021) add new layers of complexity to the molecular mechanism underlying this already complex disease. Indeed, in future studies it might be recognized that the depletion of these proteins will not only affect IFN-I production but potentially genome stability itself, which will need to be considered.

Evidence suggesting that IFN-I in FA arises from the cGAS-STING pathway is accumulating. However, given the findings on mtRNA released after mtDNA damage (Ghosh et al., 2018), and the accumulation of RTE (Zhao et al., 2018) and R-loops in FA deficient cells (Garcia-Rubio et al., 2015), it is possible that RLRs also contribute to IFN-I production in FA. In such cases, both self-DNA and self-RNA would have the capacity to further induce the expression of RLRs and cGAS, since these PRRs are themselves IFN-stimulated genes (Rusinova et al., 2013). Elevated levels of these PRRs could then sensitize the cells even further to self-nucleic acids sensing, which would create a pathological feedback loop. However, similar to the role of cGAS in inhibiting HR, RIG-I has recently been shown to suppress NHEJ (Guo et al., 2021). Given that FA deficient cells are more prone to repair their DNA using the NHEJ pathway rather than the HR pathway, these data suggest that in the context of FA, RIG-I would not play this NHEJ inhibitory role. These findings reflect the complex integration of immune signalling and DNA repair pathway. Thus, it is expected that RLRs and cGAS might have different roles and be differently regulated depending on the cellular context promoted by specific FA gene mutations.

Another important question is whether defect in each one of the 23 FA proteins have similar consequences on IFN-I. Research suggests that FA genes downstream of ID2 complex have a crucial role in orchestrating the HR response through the recruitment of endonucleases and other factors that will allow strand invasion and recombination. Loss of BRCA related genes contributes to a switch from HR to NHEJ and consequently facilitates cytosolic self-DNA accumulation and thus cGAS activation. Nonetheless, whether FA proteins upstream of ID2 complex will have the same consequences remains to be demonstrated specifically in cells with functional cell cycle checkpoint. In fact, in the case of the structure-specific endonuclease MUS81, a study shows that its activity was critical to the accumulation of cytosolic DNA. Consequently, if defects in specific FA genes prevent the action of endonucleases, inflammatory signals will not be produced.

FA is a phenotypically heterogenous disease, and while we can easily expect a role for IFN-I in bone marrow failure and cancer, its importance in congenital abnormalities is less clear. Contrary to AGS, IFN-I in FA is not expected to be produced at high levels and may be restricted to specific cell populations. In AGS, degradation of nucleic acids is impaired and, thus, self-nucleic acids accumulate without the need for cells to display high levels of DNA damage. In FA however, only cells that proliferate in the presence of DNA damage or with high demands in mitochondria clearance for instance are expected to display high levels of cytosolic DNA. As we report in this review, IFN-I in FA could be produced by distinct molecular mechanisms. As such, it would be of interest to investigate what the predominant mechanism in different cell populations is.

## Future Perspectives

Many questions warrant further investigations that will necessitate the generation of new mouse models. Generation of complete knock-out and subsequently tissue-specific knock-out for the different players implicated in IFN-I signaling in FA (IFNAR, cGAS, STING and RIG-I). These new models should reveal not only if IFN-I is implicated in the pathophysiology of FA, but also in which cell population the production of IFN-I originates. Given the challenge associated with a complete inhibition of the IFN-I signaling, it is difficult to predict how Jak inhibitor would work in FA patients since we expect IFN-I levels to be lower than in AGS. To achieve better clinical results, other therapeutic targets, such as cGAS or STING, might need to be considered. On a positive note, these future researches have the potential to uncover new therapeutic opportunities for FA patients and hence contribute to expand their life expectancy.

## Author Contributions

KL wrote sections of the first draft and edited the manuscript. SS designed, drew the schematic and edited the manuscript. GP designed, wrote and edited the manuscript. All authors approved the final version of the manuscript.

## Funding

Fonds de Recherche du Québec (FRSQ) – Santé (35071 to GP); Chaire de Recherche Junior UQTR (GP); CERMO-FC (GP); the Canadian Foundation for Innovation (CFI-40780 to GP); MITACS Globalink scholarship (UQTR-97834 to SS).

## Conflict of Interest

The authors declare that the research was conducted in the absence of any commercial or financial relationships that could be construed as a potential conflict of interest.

## Publisher’s Note

All claims expressed in this article are solely those of the authors and do not necessarily represent those of their affiliated organizations, or those of the publisher, the editors and the reviewers. Any product that may be evaluated in this article, or claim that may be made by its manufacturer, is not guaranteed or endorsed by the publisher.
